# Supplementary material for: High-Performance Multilevel and Ambipolar Nonvolatile Organic Transistor Memory Using Small-Molecule SFDBAO and PS as Charge Trapping Elements
Source: Nanomaterials (Basel). 2025 Jul 10;15(14):1072. doi: 10.3390/nano15141072 (PMC12300832; doi:10.3390/nano15141072)
Supplement: Supplementary file 1 [file nanomaterials-15-01072-s001.zip › nanomaterials-3702637-supplementary.pdf]

nanomaterials

Supporting Information

High-performance Multilevel and Ambipolar Nonvolatile Organic Transistor Memory using Small Molecule SFDBAO and PS as Charge Trapping Elements

Lingzhi Jin [1,†], Wenjuan Xu [2,†], Yangzhou Qian [1], Tao Ji [1], Kefan Wu [1], Liang Huang [1], Feng Chen [1], Nanchang Huang [1], Shu Xing [1], Zhen Shao [2], Wen Li [2], Yuyu Liu [1,*] and Linghai Xie [2,*]

1 School of Electronics Information Engineering & School of Integrated Circuits, Nanjing ~~University~~ of Industry Technology, Nanjing 210023, China
2 State Key Laboratory for Organic Electronics and Information Displays & Institute of Advanced Materials (IAM), Nanjing University of Posts and Telecommunications (NJUPT), Nanjing 210023, China

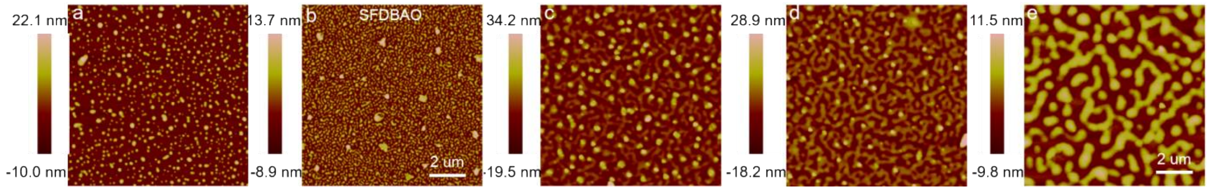

Figure S1. AFM topographic images (10 μm × 10 μm) of thermally evaporated SFDBAO thin films on SiO_2/Si substrates under different deposition conditions. The evaporation rate and time of SFDBAO thin films are (a) 0.25 Å/s, 125 s, (b) 0.25 Å/s, 250 s, (c) 0.25 Å/s, 375 s, (d) 0.25 Å/s, 500 s and (e) 0.15 Å/s, 400 s, respectively. .

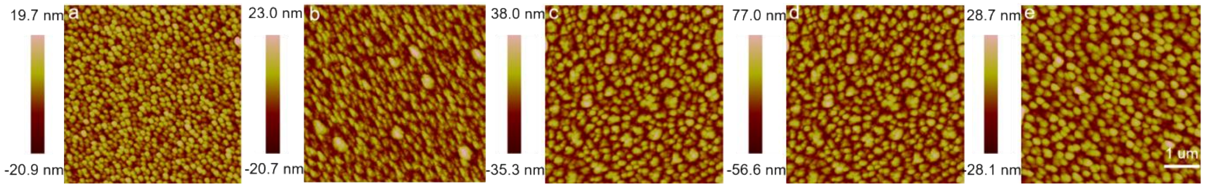

Figure S2. AFM topographic images (5 μm × 5 μm) of pentacene thin films deposited on different SFDBAO thin films. The evaporation rate and time of SFDBAO thin films are (a) 0.25 Å/s, 125 s, (b) 0.25 Å/s, 250 s, (c) 0.25 Å/s, 375 s, (d) 0.25 Å/s, 500 s and (e) 0.15 Å/s, 400 s, respectively. .

Table S1. Performance for OFET memory under various film deposition conditions.

Evaporation Rate [Å/s]	Evaporation Time [s]	Mobility [$cm^2 V^{-1} s^{-1}$]	Threshold Voltage [V]	Negative window [V]	I_{ON}/I_{OFF}
0.25	125	1.54×10^{-3}	-2.18	25	1.86×10^{2}
0.25	250	6.25×10^{-3}	-6.55	45	2.62×10^{3}
0.25	375	4.97×10^{-4}	-6.96	50	3.73×10^{3}
0.25	500	9.28×10^{-4}	-23	63	1.70×10^{2}
0.15	400	3.55×10^{-4}	-7.42	42	6.80×10^{3}

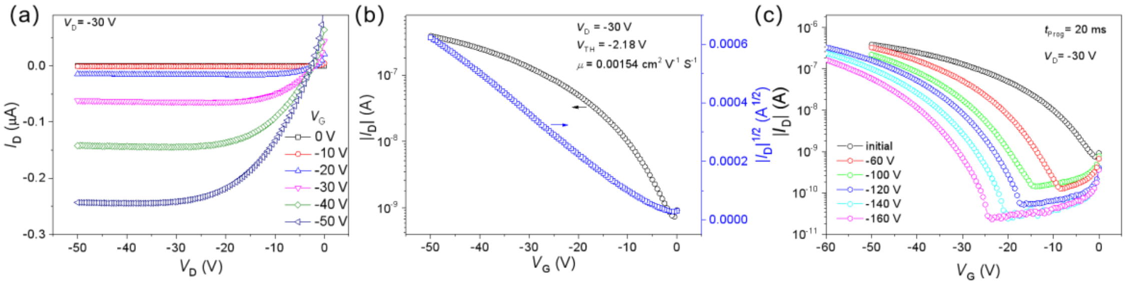

Figure S3. Output (a), Transfer Characteristics (b) and Transfer curves under different programming voltage (c) of SFDBAO-based ONVM. The evaporation rate and time of SFDBAO thin films are 0.25 Ǻ/s and 125 s, respectively.

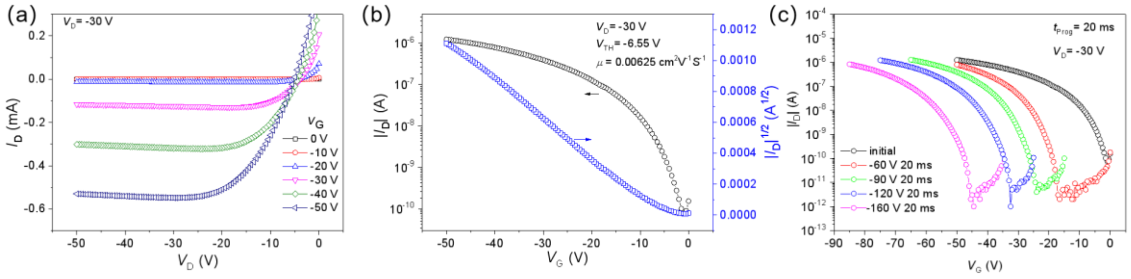

Figure S4. Output (a), Transfer Characteristics (b) and Transfer curves under different programming voltage (c) of SFDBAO-based ONVM. The evaporation rate and time of SFDBAO thin films are 0.25 Ǻ/s and 250 s, respectively.

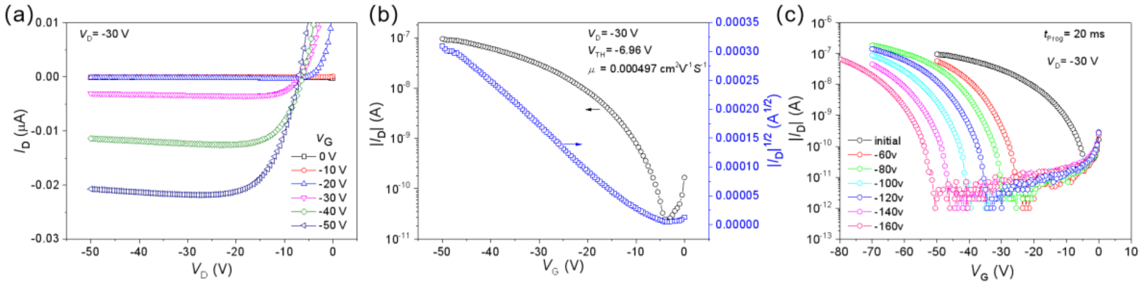

Figure S5. Output (a), Transfer Characteristics (b) and Transfer curves under different programming voltage (c) of SFDBAO-based ONVM. The evaporation rate and time of SFDBAO thin films are 0.25 Ǻ/s and 375 s, respectively.

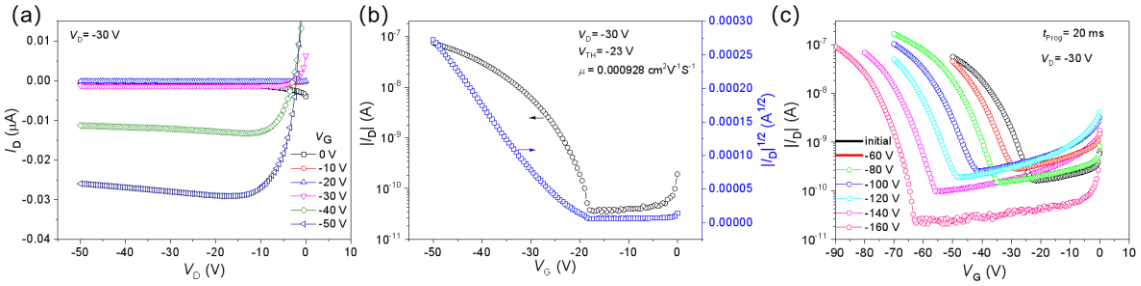

Figure S6. Output (a), Transfer Characteristics (b) and Transfer curves under different programming voltage (c) of SFDBAO-based ONVM. The evaporation rate and time of SFDBAO thin films are 0.25 Ǻ/s and 500 s, respectively.

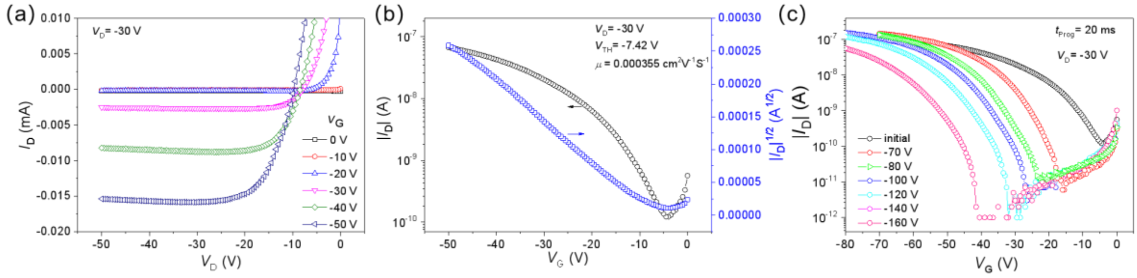

Figure S7. Output (a), Transfer Characteristics (b) and Transfer curves under different programming voltage (c) of SFDBAO-based ONVM. The evaporation rate and time of SFDBAO thin films are 0.15 Å/s and 400 s, respectively.

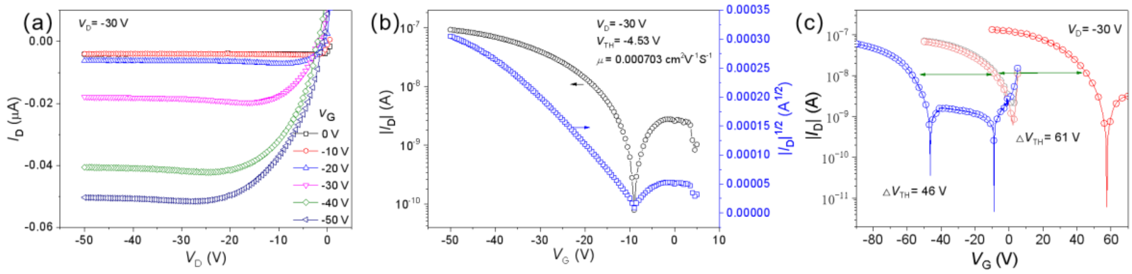

Figure S8. Output (a), Transfer Characteristics (b) and Transfer curves under different programming voltage (c) of the SFDBAO-based ONVM. SFDBAO thin film is made by spin-coating method.

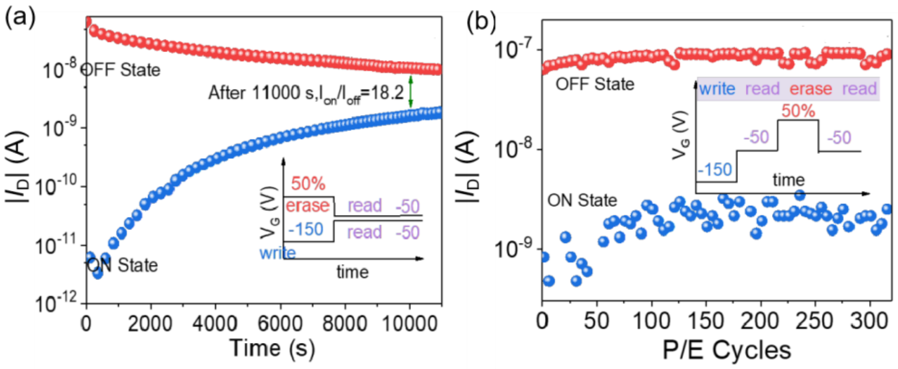

Figure S9. Retention characteristics of the SFDBAO-based ONVM. (a) ONVM after the writing and erasing operation maintain over 1.1×10⁴ s for Hole trapping mode. (b) Endurance characteristics of the SFDBAO-based ONVM over a series of programming, reading, and erasing processes for Hole trapping mode. SFDBAO thin film is made by spin-coating method.

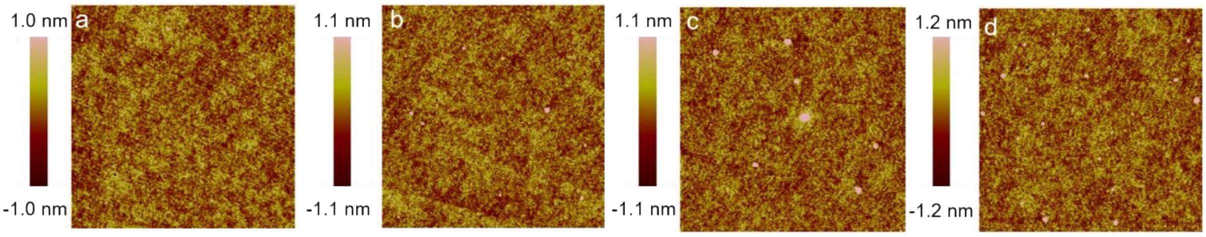

Figure S10. AFM topographic images (10 μm × 10 μm) of SFDBAO/PS composite films with SFDBAO blend ratio of (a) 0, (b) 5%, (c) 20% and (d) 25%, respectively.

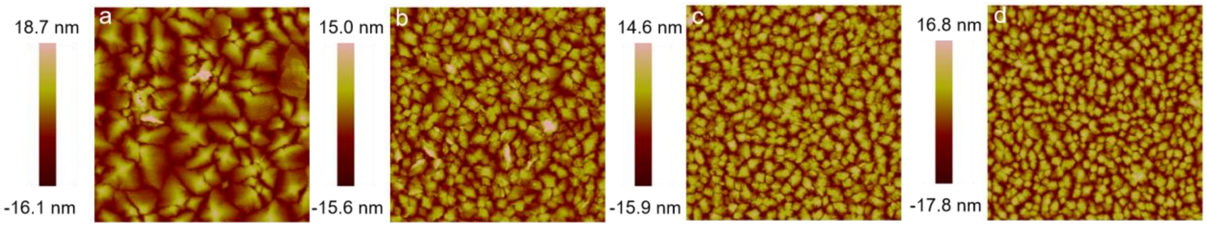

Figure S11. AFM topographic images (5 μm × 5 μm) of pentacene thin films deposited on SFDBAO/PS composite films with SFDBAO blend ratio of (a) 0, (b) 5%, (c) 20% and (d) 25%, respectively.

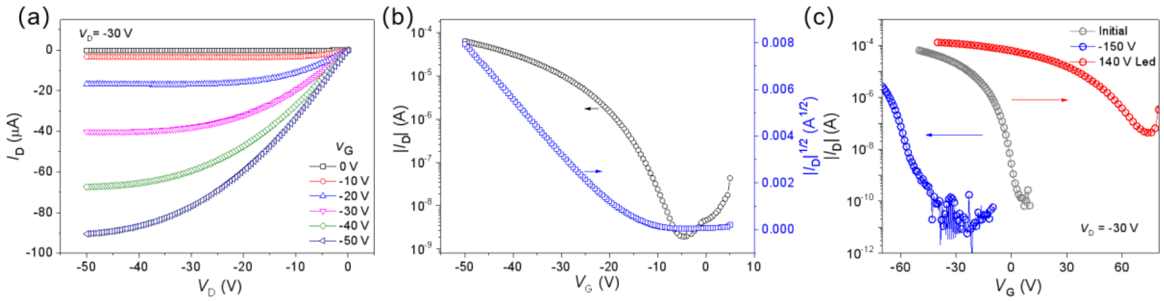

Figure S12. (a) Output characteristics, (b) Transfer characteristics and (c) Reversible shifts in transfer curves of transistor memory devices based on 5 % SFDBAO/PS composite charge trapping layer.

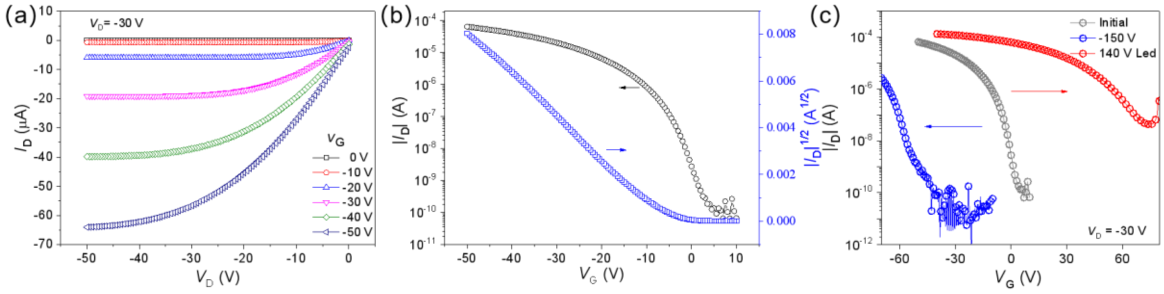

Figure S13. (a) Output characteristics, (b) transfer characteristics and (c) reversible shifts in transfer curves of transistor memory devices based on 15% SFDBAO/PS composite charge trapping layer.

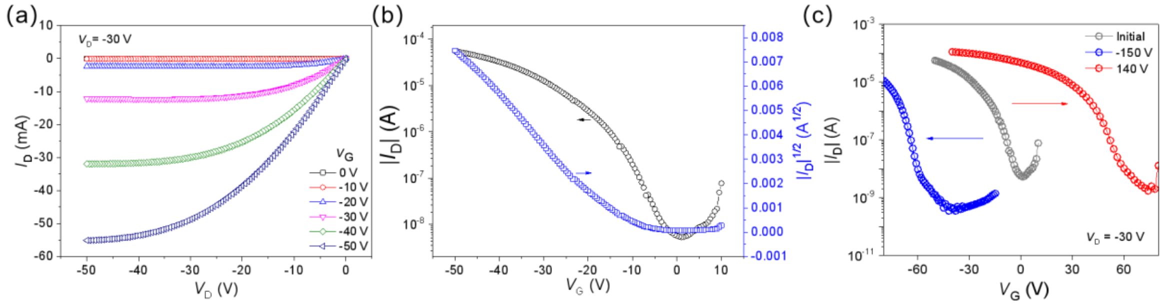

Figure S14. (a) Output characteristics, (b) Transfer characteristics and (c) Reversible shifts in transfer curves of transistor memory devices based on 20% SFDBAO/PS composite charge trapping layer.

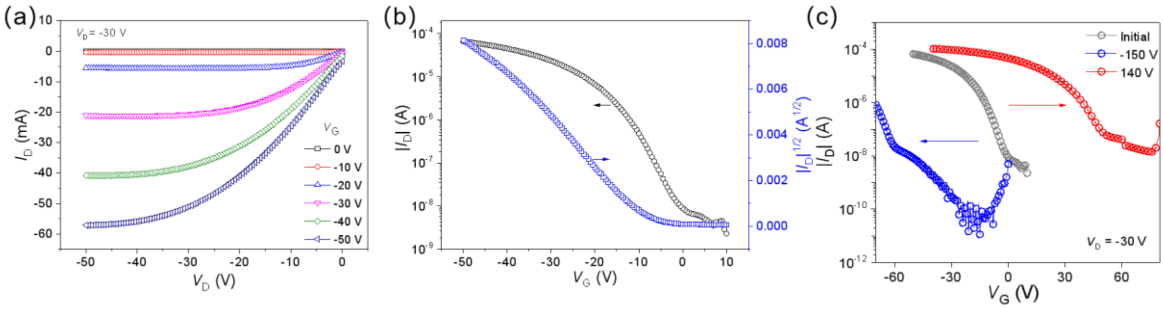

Figure S15. (a) Output characteristics, (b) Transfer characteristics and (c) Reversible shifts in transfer curves of transistor memory devices based on 25% SFDBAO/PS composite charge trapping layer.

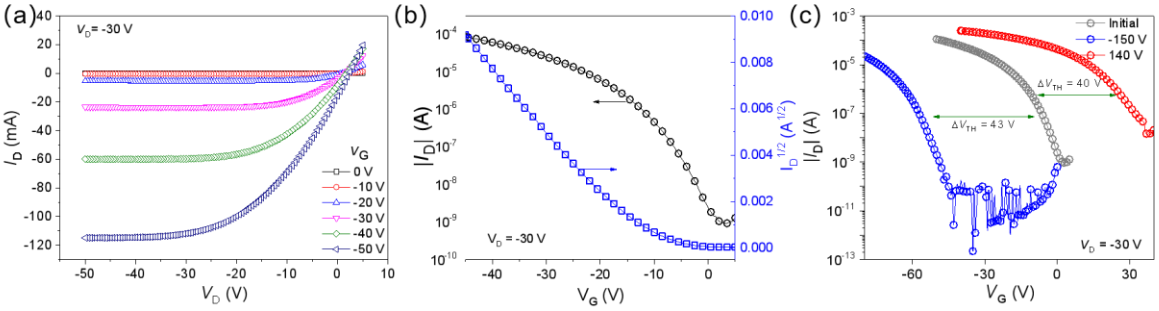

Figure S16. (a) Output characteristics, (b) Transfer characteristics and (c) Reversible shifts in transfer curves of transistor memory devices based on 10% C_{60}/PS composite charge trapping layer.

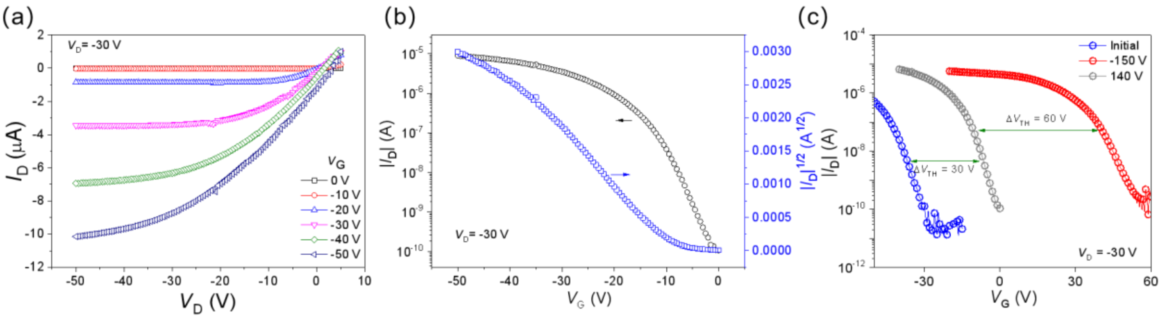

Figure S17. (a) Output characteristics, (b) Transfer characteristics and (c) Reversible shifts in transfer curves of transistor memory devices based on 10% Alq_3/PS composite charge trapping layer.

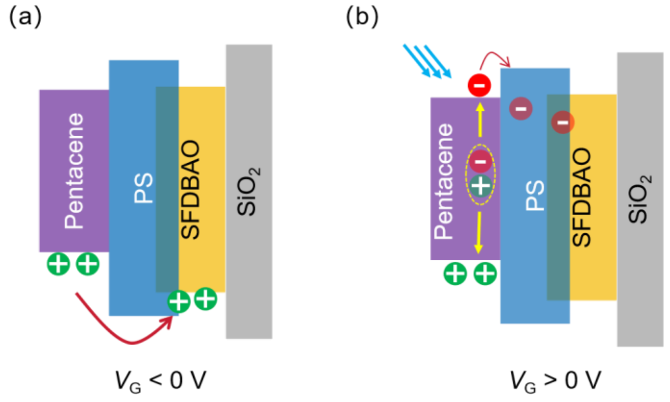

Figure S18. Schematic of (a) holes trapping in dark and (b) electrons trapping under lighting conditions.
